# Supplementary material for: Role of Lipids and Divalent Cations in Membrane Fusion Mediated by the Heptad Repeat Domain 1 of Mitofusin
Source: Biomolecules. 2023 Sep 2;13(9):1341. doi: 10.3390/biom13091341 (PMC10527301; doi:10.3390/biom13091341)
Supplement: Supplementary file 1 [file biomolecules-13-01341-s001.zip › biomolecules-2552169-original images/Gel 1_Figure 2a.pptx]

## Slide 1
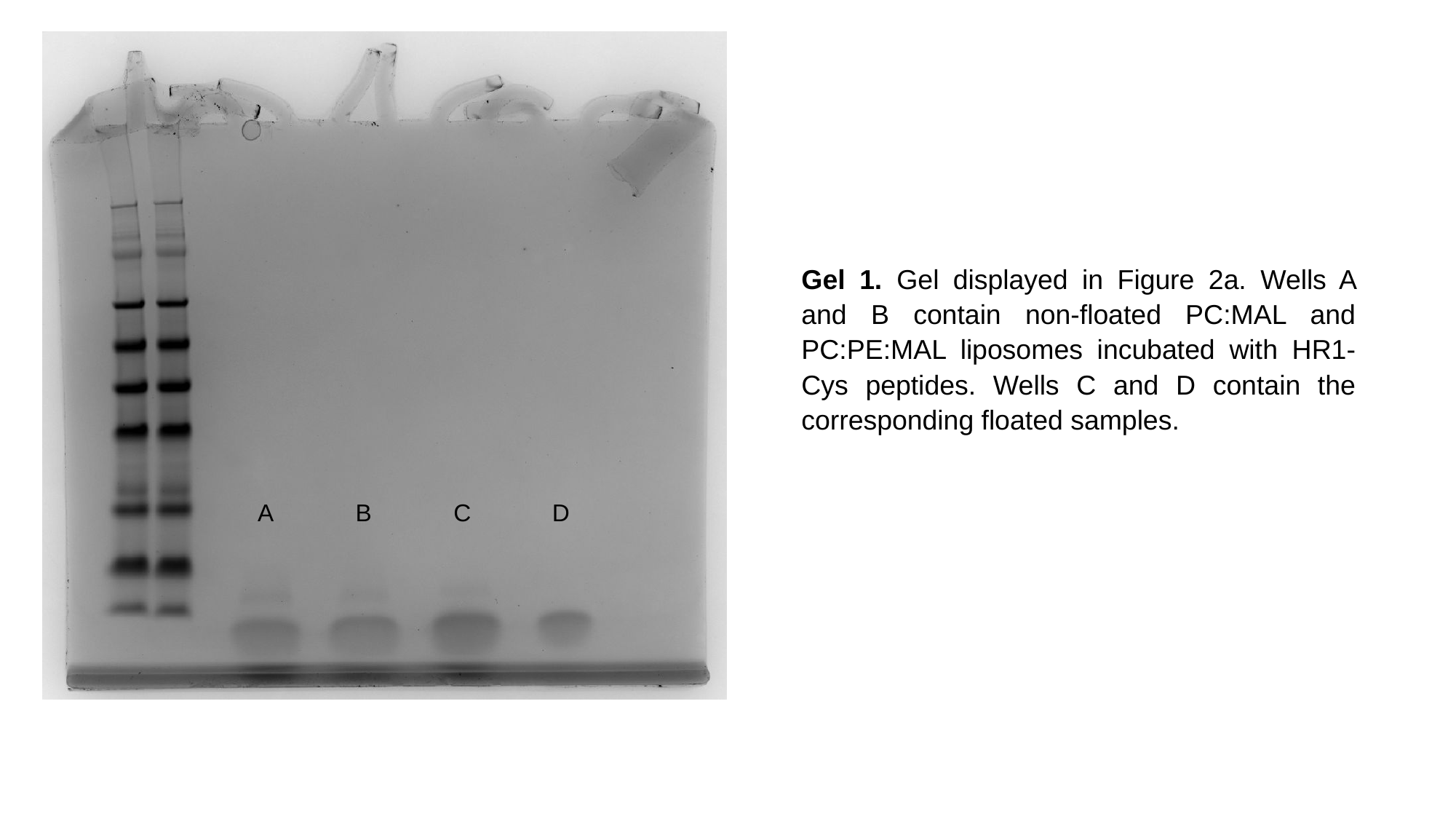

Gel 1. Gel displayed in Figure 2a. Wells A and B contain non-floated PC:MAL and PC:PE:MAL liposomes incubated with HR1-Cys peptides. Wells C and D contain the corresponding floated samples.
D
A
B
C
