# Supplementary material for: Role of Lipids and Divalent Cations in Membrane Fusion Mediated by the Heptad Repeat Domain 1 of Mitofusin
Source: Biomolecules. 2023 Sep 2;13(9):1341. doi: 10.3390/biom13091341 (PMC10527301; doi:10.3390/biom13091341)
Supplement: Supplementary file 1 [file biomolecules-13-01341-s001.zip › biomolecules-2552169-original images/Gel 3_Figure S3.pptx]

## Slide 1
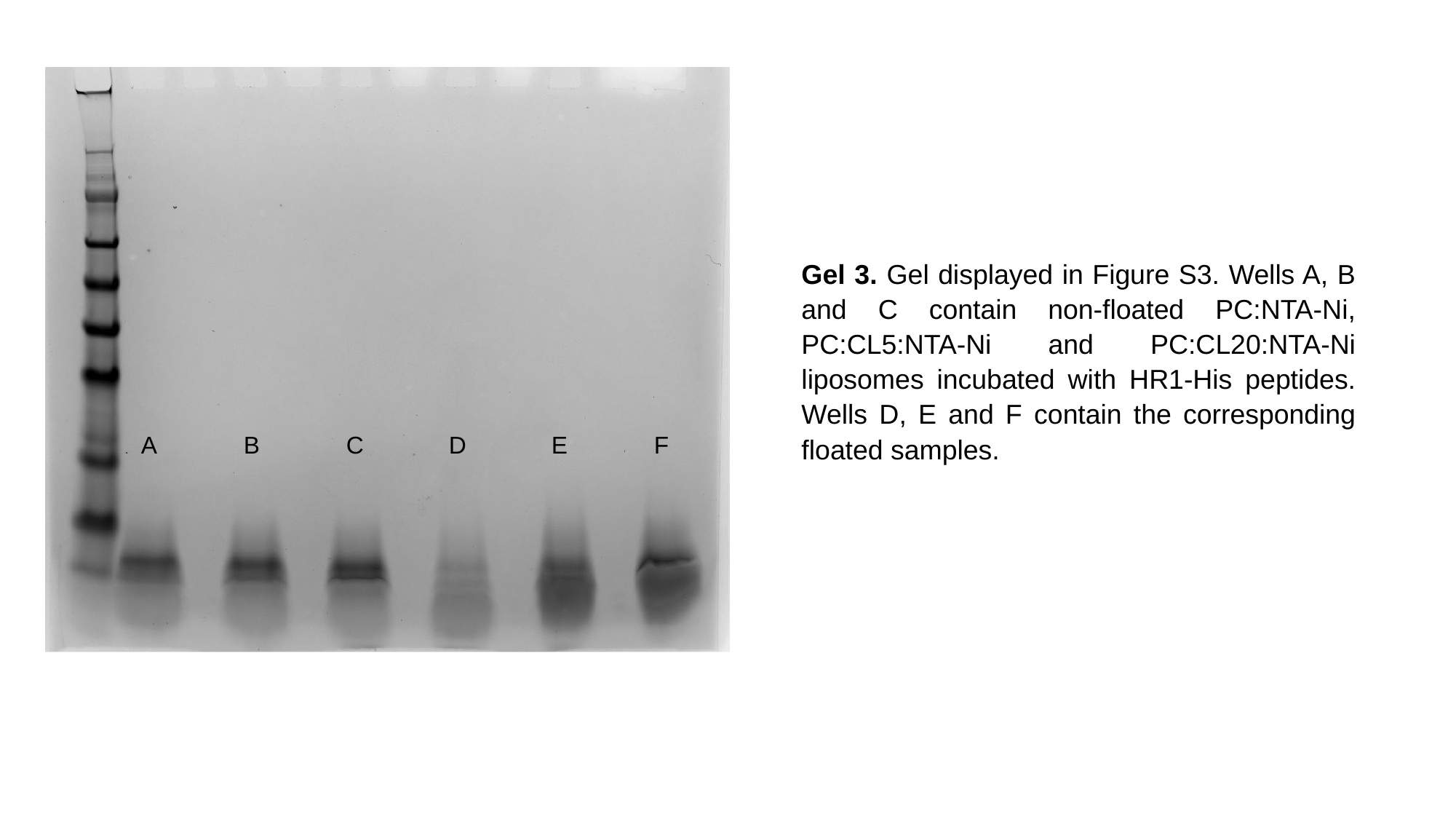

Gel 3. Gel displayed in Figure S3. Wells A, B and C contain non-floated PC:NTA-Ni, PC:CL5:NTA-Ni and PC:CL20:NTA-Ni liposomes incubated with HR1-His peptides. Wells D, E and F contain the corresponding floated samples.
F
D
E
A
B
C
