# Supplementary material for: Role of Lipids and Divalent Cations in Membrane Fusion Mediated by the Heptad Repeat Domain 1 of Mitofusin
Source: Biomolecules. 2023 Sep 2;13(9):1341. doi: 10.3390/biom13091341 (PMC10527301; doi:10.3390/biom13091341)
Supplement: Supplementary file 1 [file biomolecules-13-01341-s001.zip › biomolecules-2552169-original images/Gel 4_replicate 1_Figure 2b.pptx]

## Slide 1
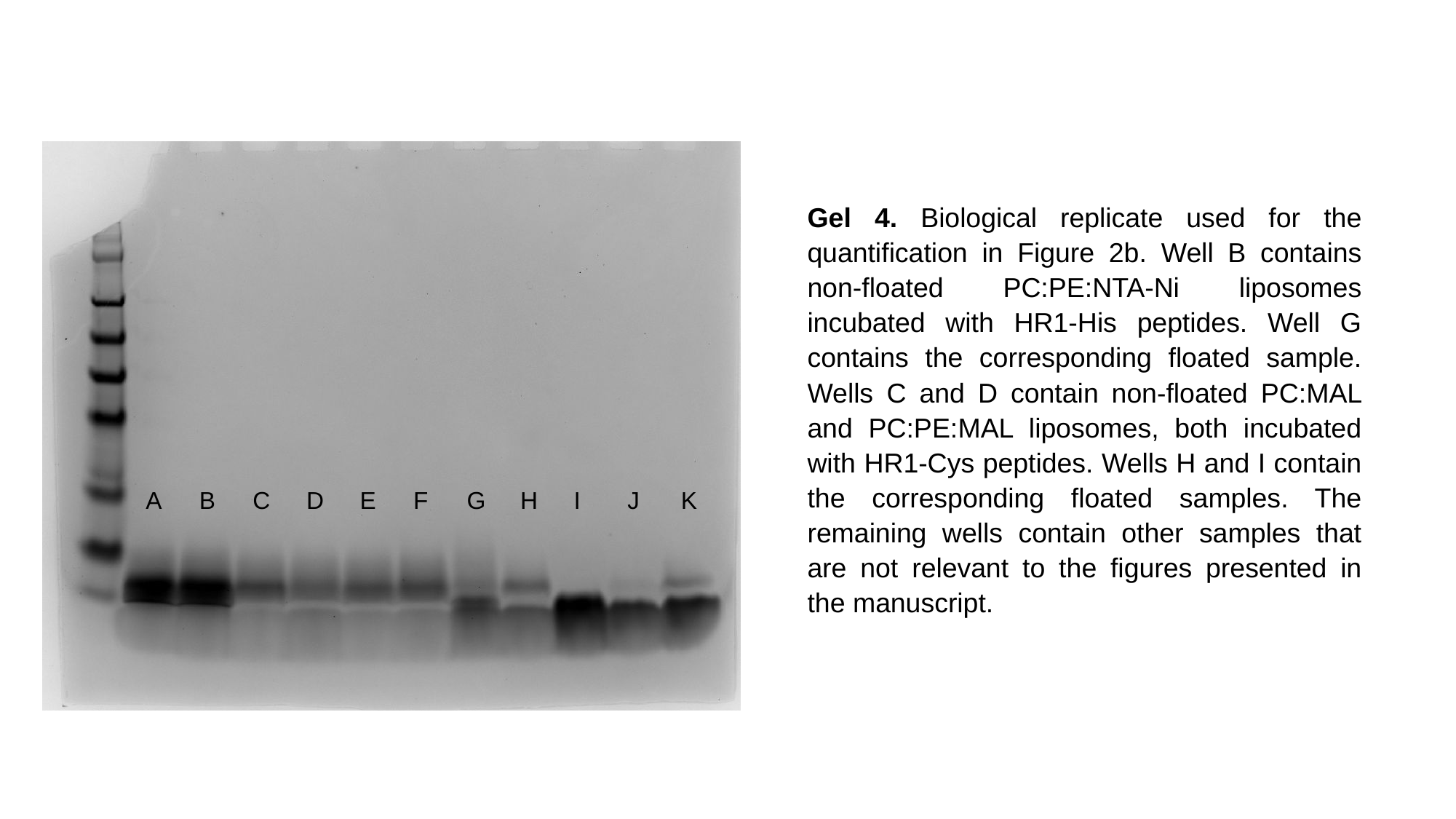

Gel 4. Biological replicate used for the quantification in Figure 2b. Well B contains non-floated PC:PE:NTA-Ni liposomes incubated with HR1-His peptides. Well G contains the corresponding floated sample. Wells C and D contain non-floated PC:MAL and PC:PE:MAL liposomes, both incubated with HR1-Cys peptides. Wells H and I contain the corresponding floated samples. The remaining wells contain other samples that are not relevant to the figures presented in the manuscript.
J
K
F
G
H
I
D
E
A
B
C
