# Supplementary material for: Role of Lipids and Divalent Cations in Membrane Fusion Mediated by the Heptad Repeat Domain 1 of Mitofusin
Source: Biomolecules. 2023 Sep 2;13(9):1341. doi: 10.3390/biom13091341 (PMC10527301; doi:10.3390/biom13091341)
Supplement: Supplementary file 1 [file biomolecules-13-01341-s001.zip › biomolecules-2552169-supplementary.pdf]

Supplementary Materials

Role of Lipids and Cations in Membrane Fusion Mediated by the Heptad Repeat Domain 1 of Mitofusin

Anaïs Vlieghe [1], Kristina Niort [1], Hugo Fumat [1], Jean-Michel Guigner [2], Mickaël M. Cohen [3] and David Tareste [1,*]

[1] Université Paris Cité, Institute of Psychiatry and Neuroscience of Paris (IPNP), Inserm UMR-S 1266, Team Membrane Traffic in Healthy & Diseased Brain, 75014 Paris, France
[2] Sorbonne Université, Institut de Minéralogie, de Physique des Matériaux et de Cosmochimie (IMPMC), CNRS UMR 7590, MNHN, IRD UR 206, 75005 Paris, France
[3] Sorbonne Université, Institut de Biologie Physico-Chimique (IBPC), CNRS UMR 8226, Laboratoire de Biologie Moléculaire et Cellulaire des Eucaryotes, 75005 Paris, France
* Correspondence: david.tareste@inserm.fr

Figure S1

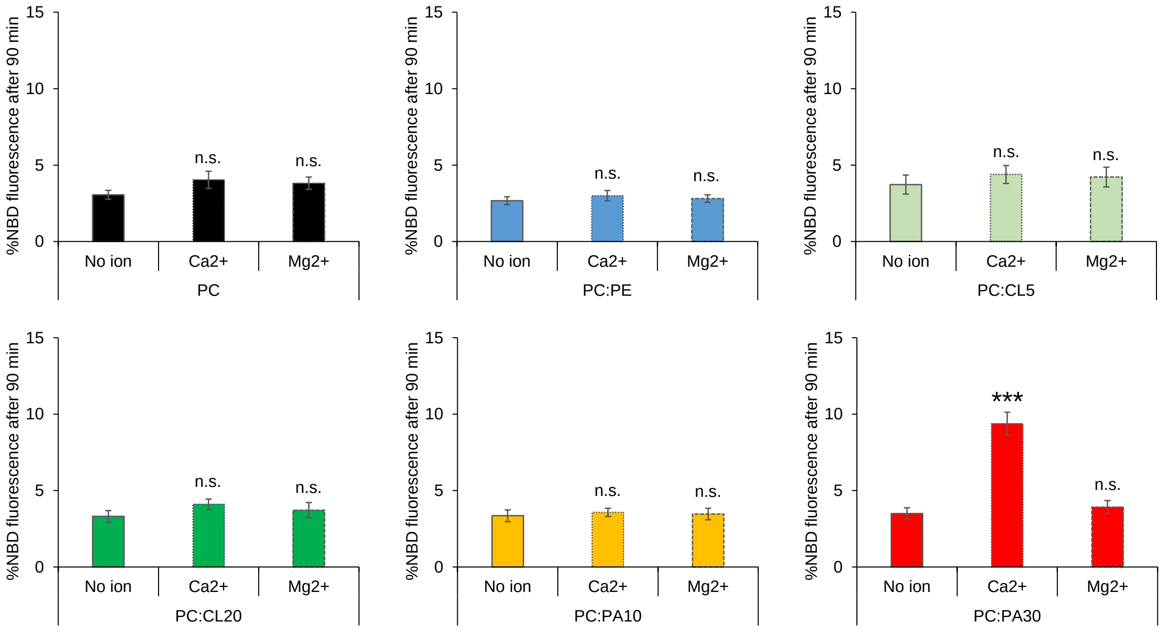

Figure S1. Average extent of lipid mixing after a 90-min reaction in fusion control experiments with liposomes functionalized with NTA-Ni lipids, where buffer alone was used instead of HR1. The experimental conditions are the same as those in Figures 1-4. Data represent the average of n=3-20 independent experiments, with error bars indicating standard errors of the mean. Statistical comparisons were performed using two-sample t-tests against the condition without ions (n.s. $p > 0.05$; *** $p < 0.001$).

Figure S2

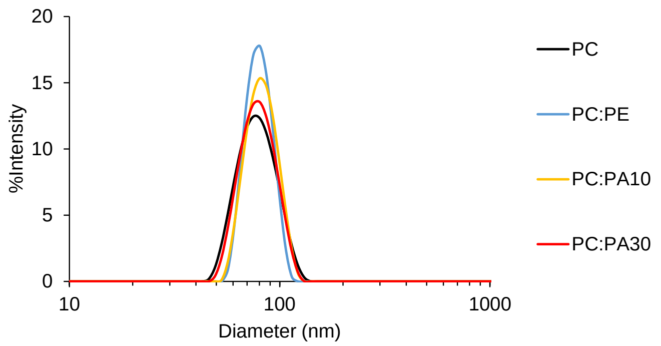

Figure S2. Representative size distribution of liposomes functionalized with NTA-Ni lipids, determined by multi-angle dynamic light scattering, as a function of their lipid composition. Lipid compositions are the same as those in Figures 1-4.

Figure S3

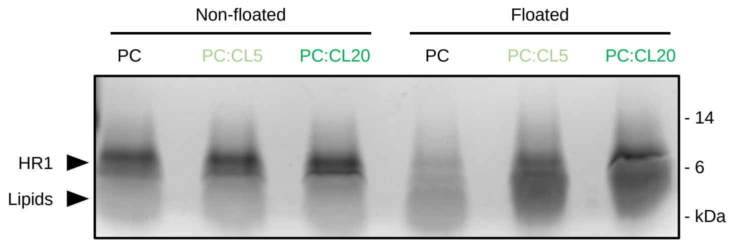

Figure S3. Liposomes with the same lipid compositions as in Figure 3 were incubated with HR1-His$_6$ peptides (500 μM lipids and 25 μM peptides) at 37°C for 1 hour. The reaction mixes were separated using a discontinuous nycodenz gradient to distinguish HR1-bound liposomes from unbound HR1. Protein and lipid recoveries in the floated samples were estimated by SDS-PAGE stained with Coomassie upon comparison with the non-floated samples.

Figure S4

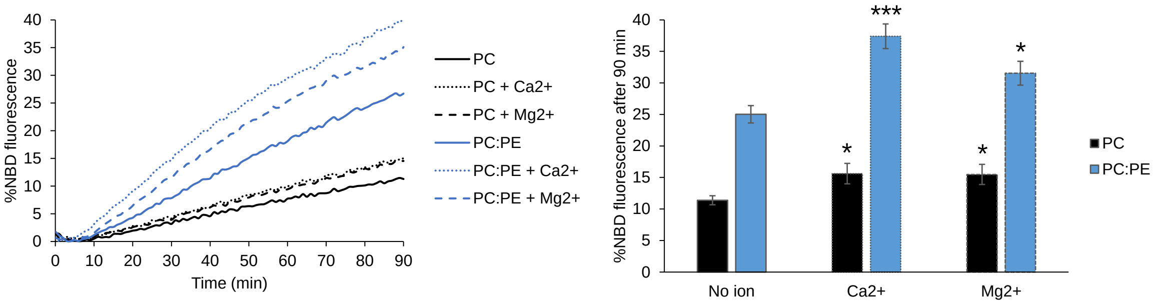

(**a**) (**b**)

Figure S4. (**a**) Representative kinetics of a FRET-based lipid mixing assay between liposomes containing 5 mol% NTA-Ni lipids in their membrane, along with 95 mol% PC (black) or 65 mol% PC and 30 mol% PE (blue). The fusion reaction was initiated by adding HR1-His$_6$ peptides at t=0 in the absence or presence of the divalent cations Ca^{2+} or Mg^{2+} (500 µM lipids, 25 µM peptides, and 1 mM cations). Control experiments with buffer alone instead of HR1 are presented in Figure S1. (**b**) Average extent of lipid mixing observed after a 90-min period, based on data from n=6-21 independent kinetics experiments, similar to the one presented in panel a. The error bars represent the standard errors of the mean. Statistical comparisons were performed using two-sample t-tests against the condition without ions and with the same lipid composition (* $p < 0.05$; *** $p < 0.001$).
